# Schizophrenia-related dysbindin-1 gene is required for innate immune response and homeostasis in the developing subventricular zone

**DOI:** 10.1038/s41537-018-0057-5

**Published:** 2018-07-23

**Authors:** Abeer R. Al-Shammari, Sanjeev K. Bhardwaj, Ksenia Musaelyan, Lalit K. Srivastava, Francis G. Szele

**Affiliations:** 1 Department of Physiology, Anatomy and Genetics, University of Oxford, Oxford, UK; 2 Research and Development, Qatar Research Leadership Program, Qatar Foundation, Doha, Qatar; 3 Neurological Disorders Research Center, Qatar Biomedical Research Institute, Hamad Bin Khalifa University, Qatar Foundation, Doha, Qatar; 4 Douglas Mental Health University Institute, McGill University, Montreal, Canada

## Abstract

Schizophrenia is a neurodevelopmental disorder likely caused by environmental and genetic risk factors but functional interactions between the risk factors are unclear. We tested the hypothesis that dysbindin-1 (*Dtnbp1*) gene mutation combined with postnatal exposure to viral mimetic polyI:C results in schizophrenia-related behavioural changes in adulthood, and mediates polyI:C-induced inflammation in the subventricular zone (SVZ). Adult Sandy (Sdy, *Dtnbp1* mutant) mice given early postnatal polyI:C injections displayed reduced prepulse inhibition of startle, reduced locomotion and deficits in novel object recognition. PolyI:C induced a canonical immune response in the SVZ; it increased mRNA expression of its toll-like receptor 3 (*Tlr3*) and downstream transcription factors *RelA* and *Sp1*. PolyI:C also increased SVZ *Dtnbp1* mRNA expression, suggesting dysbindin-1 regulates immune responses. Dysbindin-1 loss in Sdy mice blocked the polyI:C-induced increases in mRNA expression of *Tlr3*, *RelA* and *Sp1* in the SVZ. *Dtnbp1* overexpression in SVZ-derived Sdy neurospheres rescued *Tlr3*, *RelA* and *Sp1* mRNA expression supporting a functional interaction between dysbindin-1 and polyI:C-induced inflammation. Immunohistochemistry showed higher Iba1+ immune cell density in the SVZ of Sdy mice than in WT postnatally. PolyI:C did not alter SVZ Iba1+ cell density but increased CD45+/Iba1− cell numbers in the SVZ of Sdy mice. Finally, polyI:C injections in Sdy, but not WT mice reduced postnatal and adult SVZ proliferation. Together, we show novel functional interactions between the schizophrenia-relevant dysbindin-1 gene and the immune response to polyI:C. This work sheds light on the molecular basis for amplified abnormalities due to combined genetic predisposition and exposure to environmental schizophrenia risk factors.

## Introduction

Multiple genetic and environmental factors increase the risk for developing schizophrenia.^1^ For example, genetic variation in dysbindin-1 (*DTNBP1*) is associated with schizophrenia as confirmed in various populations^2,3^ and schizophrenia patients with normal *DTNBP1* sequence have reduced dysbindin-1 expression in the brain.^4–7^ Environmental insults such as viral infections during neurodevelopment increase the risk of developing schizophrenia.^8^ Polyinosinic-polycytidylic acid (polyI:C) is an immunostimulant commonly used to mimic viral infection during early development in animal models of schizophrenia.^9,10^ PolyI:C is a synthetic analog of double-stranded RNA (dsRNA) and it is specifically recognized by toll-like receptor 3 (Tlr3), which, in turn activates the transcription factor RelA resulting in strong innate immune responses.^11^ RelA and another transcription factor, Sp1, transactivate each other during viral infections^12–15^ and both RelA^16^ and Sp1^17,18^ are implicated in schizophrenia.

Maternal inflammation during pregnancy is commonly used as an animal model of schizophrenia, however in our study we used polyI:C injections during postnatal day 5 (P5) to P9 for several reasons. Based on a mathematical model for translating neurodevelopmental events across mammalian species (http://translatingtime.org),^19^ brain growth and neurogenesis (in the striatum, limbic system and also the whole brain) of the mouse at P5 (postconception (PC) day 23.5) and P9 (PC day 27.5) translate to PC day 173 and 236, respectively, in the human which spans the second to third trimester.^19^ Of interest, these times in mice and human lie within a peak period of brain growth, gliogenesis, increasing axonal growth and dendritic density and immune system development.^19,20^ In addition, we used postnatal mice in order to study the direct impact of polyI:C on the developing postnatal brain and avoid the complications of maternal-fetal immune crosstalk. Furthermore, a number of epidemiological studies show increased risk of developing schizophrenia after early postnatal infection.^8^ Interestingly, a recent study suggests that maternal infections during pregnancy contribute to the risk of childhood infections, which together increase the risk of psychosis.^21^

Epidemiological studies also suggest that gene and environment (GxE) interactions increase the risk of developing schizophrenia compared to a single factor alone.^22^ Similarly, animal models of schizophrenia with GxE interactions worsened the histological and behavioural phenotypes compared to a single risk factor.^23^ However, it is unclear how combined GxE risk factors amplify schizophrenia risk.^1^

In this study, dysbindin-1 mutant Sandy (Sdy) mice, which display schizophrenia-like behaviours and neuropathology,^3,24,25^ were injected intraperitoneally (i.p.) with polyI:C during early postnatal development and used to model a GxE interaction. We characterized phenotypes in adulthood and at P12, a few days after the final polyI:C injection in order to allow cellular phenotypes to appear, and to detect gene expression responses before they disappear. We focused on the subventricular zone (SVZ), a neurogenic stem cell niche lining the lateral ventricle close to the choroid plexus, a major route of exchange between blood and brain.^26^ SVZ cells proliferate throughout life in rodents and generate neuroblasts that move in the rostral migratory stream (RMS) to the olfactory bulbs. The SVZ also contains microglia that are constitutively more activated than in non-neurogenic regions.^27^ Therefore, the SVZ is an excellent system for elucidating not only neurodevelopmental but also inflammatory mechanisms regulated by GxE risk factors. The impact of systemic polyI:C inflammation on *Tlr3*-*RelA*-*Sp1* response of SVZ cells was studied in vivo as well as in vitro in neurospheres. We sought to determine if mutated *Dtnbp1* gene might directly influence polyI:C-induced *Tlr3*-*RelA*-*Sp1* signaling in the SVZ. Our other aims were to determine whether the GxE interaction may affect neurodevelopmental and immune cells or schizophrenia-relevant behaviours. Together, our data provide novel insights into the molecular basis of GxE amplified phenotypes.

## Results

### Postnatal polyI:C caused behavioural abnormalities relevant to schizophrenia in adult Sdy mice

**Fig. 1:**
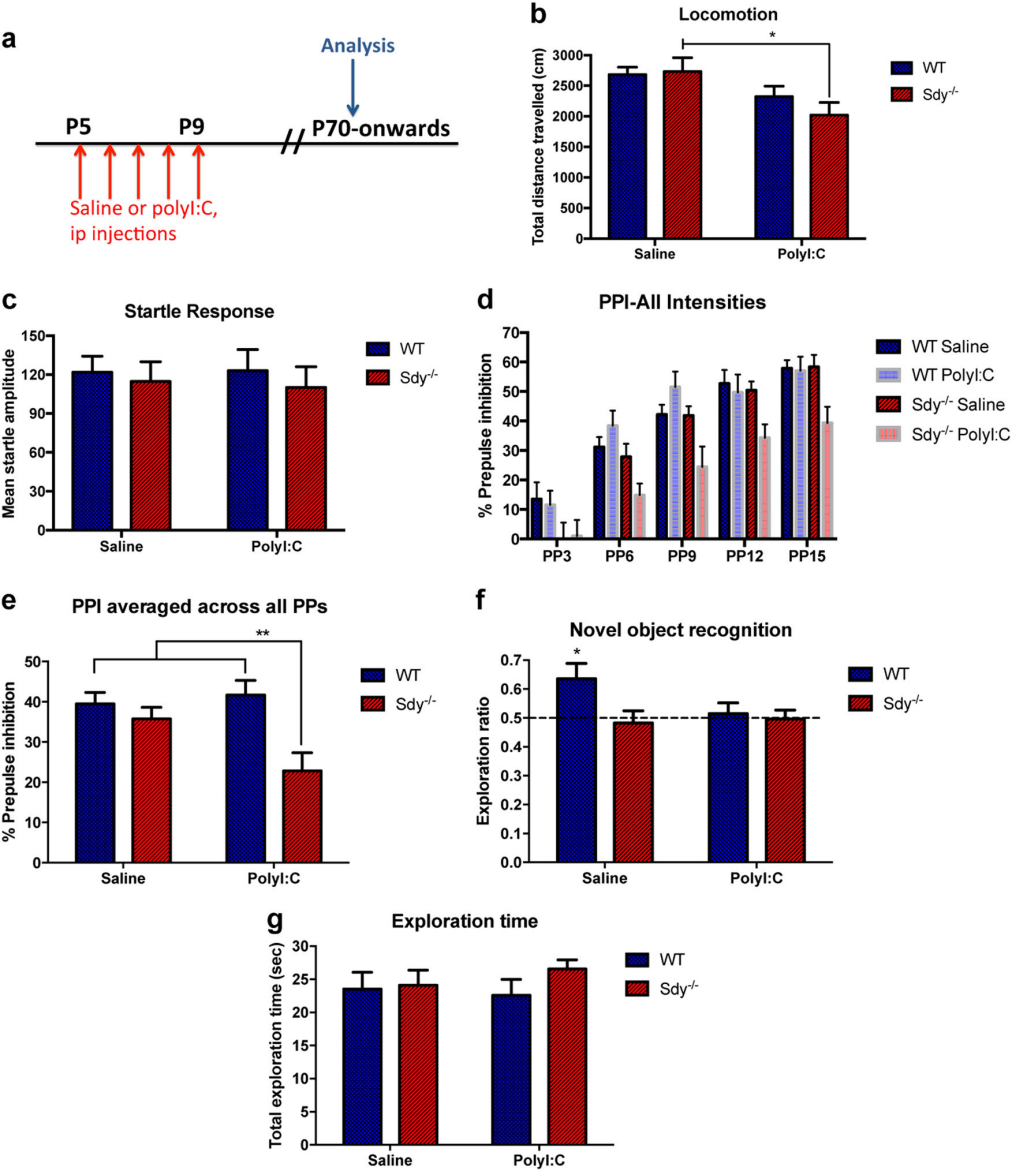
Adult Sdy mice given postnatal polyI:C displayed schizophrenia relevant behavioural abnormalities. **a** Experimental design in which mice were injected at postnatal day 5 (P5) to P9, and characterized in adulthood. **b** Spontaneous locomotor activity at P70. **c** Mean startle amplitude (baseline level) to an acoustic pulse of 120 decibels in ten trials without any prepulses. **d**, **e** Percent of prepulse inhibition (PPI) to a main pulse of 120 decibels following prepulse (PP) intensities of 3, 6, 9, 12 or 15 decibels (5 trials per each PP). **f** Novel object recognition in adulthood. **g** Exploration times. All values are mean ± s.e.m. of *N* = 12–17 per group for (**b**) and *N* = 12 per group for **c**–**g**. Data were analyzed using two-way ANOVA for (**c**, **g**) with Tukey’s post hoc test for (**b**, **e**) or Student’s two-tailed *t*-test for (**f**). *P < 0.05 and **P < 0.01

Most behavioural alterations in schizophrenia emerge in young adulthood even though genetic and environmental risk factors primarily affect perinatal neurodevelopment. We queried if schizophrenia relevant behaviours were altered in adult Sdy mice after postnatal polyI:C injections (Fig. 1a). A two-way ANOVA showed a significant main effect of polyI:C treatment on locomotor activity (*F*(1,52) = 8.793; *P* = 0.0046). Tukey’s post hoc test revealed reduced locomotion specifically in the Sdy polyI:C group compared to the Sdy saline group (*N* = 12–17; Fig. 1b). In the prepulse inhibition (PPI) test (Fig. 1d, e), two-way ANOVA revealed a significant main effect of genotype (*F*(1,44) = 10.3; *P* = 0.0025) and a significant genotype x polyI:C interaction (*F*(1,44) = 4.558; *P* = 0.0384). Further analysis using Tukey’s post hoc test revealed a significantly reduced PPI in Sdy, but not WT mice postnatally injected with polyI:C (*P* < 0.01; *N* = 12 per group; Fig. 1d, e) indicating schizophrenia-relevant sensorimotor deficits. This effect is not due to a difference in the startle response to 120 dB tone pulses between the groups (two-way ANOVA; *N* = 12 per group; *P* > 0.05; Fig. 1c). A novel object recognition test was used to assess cognitive memory. Adult WT saline mice spent longer exploring a novel than a familiar object (a value significantly different from 0.5 chance level) as determined using Student’s two-tailed *t*-test (*t*(22) = 2.54; *P* = 0.0184; *N* = 12 per group; Fig. 1f). In contrast, the other groups did not show preference for a novel object (*N* = 12 per group; Fig. 1f), suggesting deficits due to polyI:C and/or dysbindin-1 mutation. There was no difference in the time mice explored objects during the familiarization phase (Fig. 1g). These data suggest postnatal inflammation and/or dysbindin-1 mutation resulted in cognitive memory deficits in adulthood.

Furthermore, we assessed anxiety-like behaviour in adult mice using the elevated plus maze (EPM). A two-way ANOVA on the ratio of time spent in the open arm versus closed arm showed a significant main effect of polyI:C treatment (*F*(1,30) = 4.337; *P* = 0.0459) and also a significant genotype × polyI:C interaction (*F*(1,30) = 4.872; *P* = 0.0351). Further post hoc analysis revealed that postnatal polyI:C injections to Sdy mice, but not WT mice resulted in shorter time in open arms compared to saline treated Sdy (two-way ANOVA with Tukey’s test; *P* < 0.05; *N* = 7–10 per group; Supplementary Figure 1a). However, there was no difference in open arm entries across all groups (Supplementary Figure 1b). The EPM results reveal increased anxiety-like behaviour in adult Sdy mice given postnatal polyI:C.

We also tested fear acquisition to characterize fear conditioning and memory. A three-way ANOVA repeated measures showed a significant main effect of tone-shock trials (day 1) regardless of the genotype or treatment (*F*(2,66) = 142.33; *P* < 0.001; *N* = 8–11 per group; Supplementary Figure 1c). These data suggest all mice learned the fear association task equally. In the cued memory test, we found a significant genotype x polyI:C x tone trials interaction (three-way ANOVA repeated measures; *F*(1,33) = 5.51; *P* = 0.025). Further post hoc Tukey’s test revealed that polyI:C injections to Sdy, but not WT mice significantly attenuated freezing responses compared to Sdy saline mice after presentation of tone (*P* < 0.01; *N* = 8–11 per group; Supplementary Figure 1d). In the contextual memory test, we identified a main effect of polyI:C treatment (*F*(1,33) = 7.703; *P* = 0.009) and a significant genotype x polyI:C interaction using two-way ANOVA (*F*(1,33) = 8.063; *P* = 0.0077). Post hoc Tukey’s test revealed significantly attenuated freezing in Sdy polyI:C group compared to Sdy saline group (*P* = 0.0038; *N* = 8–11 per group; Supplementary Figure 1e). However, Sdy saline mice showed significantly increased freezing to the same context in comparison to WT saline mice (*P* = 0.0495; *N* = 8–11 per group; Supplementary Figure 1e). Taken together, the Sdy polyI:C model exhibited some abnormal behaviours in adulthood relevant to schizophrenia, giving the model good face validity for the positive symptoms of schizophrenia.

### PolyI:C increases dysbindin-1 expression in the SVZ and dysbindin-1 regulates the immune response to polyI:C

**Fig. 2:**
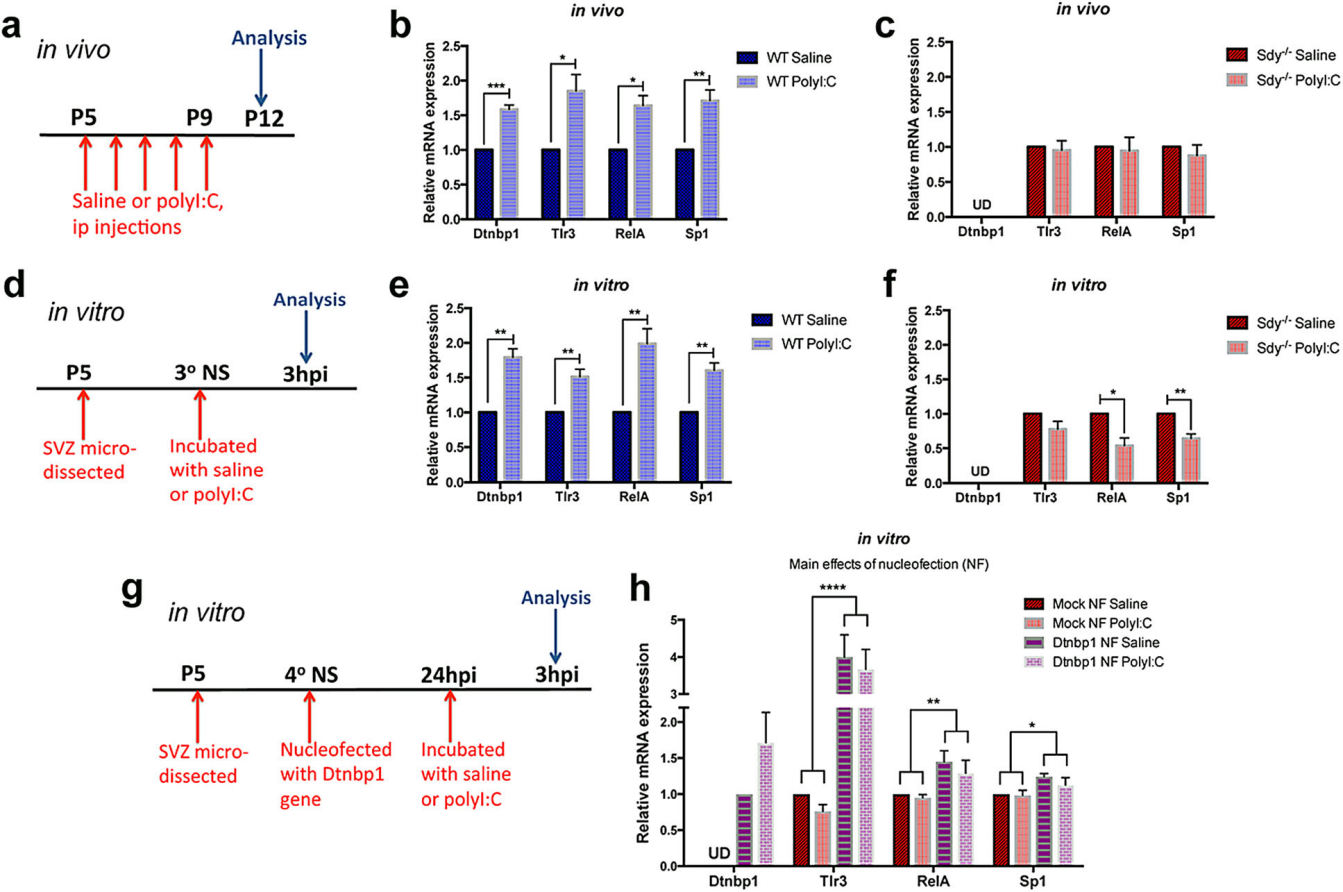
Dysbindin-1 mediates the polyI:C-induced SVZ response. **a** In vivo experimental design in which mice were injected at postnatal day 5 (P5) to P9, and SVZ analyzed at P12. **b** *Dtnbp1*, *Tlr3*, *RelA* and *Sp1* mRNA expression in the SVZ. **c** *Dtnbp1* mRNA was undetected (UD) in Sdy SVZ. PolyI:C did not increase *Tlr3*, *RelA* and *Sp1* expression in the Sdy SVZ. **d** In vitro experimental design in which tertiary SVZ neurospheres (3° NS) were analyzed 3 h post-incubation (3hpi) in saline or polyI:C. **e** *Dtnbp1, Tlr3, RelA and Sp1* mRNA expression in WT neurospheres. **f** Sdy neurospheres incubated with polyI:C did not increase *Tlr3* expression, but decreased *RelA* and *Sp1* expression. **g** Rescue experimental design in which Sdy quaternary SVZ neurospheres (4° NS) nucleofected with *Dtnbp1* gene were analyzed 3hpi in saline or polyI:C. **h** Following *Dtnbp1* nucleofection (NF) in Sdy neurospheres, *Dtnbp1* mRNA was detected and *Tlr3*, *RelA* and *Sp1* mRNA expression was increased. All values are mean ± s.e.m. from three independent experiments. Data were analyzed using Student’s two-tailed *t*-test (**b**, **c**, **e**, **f**); or two-way ANOVA (**h**). *P < 0.05; **P < 0.01; ***P < 0.001 and ****P < 0.0001

To assess dysbindin-1’s role in the response to polyI:C, we dissected the SVZ of WT and Sdy mice at P12 after saline or polyI:C injections from P5 to P9 (Fig. 2a). *Dtnbp1* mRNA was expressed in vivo in the SVZ of WT controls (Fig. 2b) but was not detected in Sdy mice, as expected (Fig. 2c). Surprisingly, *Dtnbp1* expression in the SVZ was significantly increased by polyI:C in WT mice (~1.6-fold) in comparison to WT saline controls (Student’s two-tailed *t*-test; *t*(4) = 9.721; *P* = 0.0006; *N* = 3 per group; Fig. 2b). We next asked if polyI:C could induce inflammatory gene expression in the SVZ. SVZs microdissected from the WT polyI:C group had significantly increased expression of *Tlr3* (~1.9-fold), *RelA* (~1.6-fold) and *Sp1* (~1.7-fold) mRNAs in comparison to the WT saline group as analyzed using Student’s two-tailed *t*-test (*N* = 3 per group; Fig. 2b). In contrast to WTs, in vivo polyI:C injections in Sdy mice did not increase *Tlr3*, *RelA* and *Sp1* mRNA expression compared to the Sdy saline group (Student’s two-tailed *t*-test; *N* = 3 per group; *P* > 0.05; Fig. 2c). These results suggested dysbindin-1 might regulate the polyI:C-induced SVZ response. Immunohistochemistry in the SVZ confirmed our results since polyI:C injections increased Tlr3 immunofluorescence in WT, but not in Sdy mice (Supplementary Figure 2). Thus, dysbindin-1 loss in Sdy mice blocked the canonical signalling response to polyI:C in the SVZ in vivo.

These in vivo results could have been indirect so we administered polyI:C to tertiary neurospheres which are composed of stem and progenitor cells (Fig. 2d). We detected dysbindin-1 mRNA expression in WT SVZ neurospheres incubated with saline (Fig. 2e) confirming the in vivo results and showing dysbindin-1 was not simply in axon terminals in the SVZ but expressed by stem/progenitor cells. Within 3 h of polyI:C administration to WT neurospheres, dysbindin-1 mRNA expression increased (~1.8-fold) compared to WT saline controls (Student’s two-tailed *t*-test; *t*(4) = 6.632; *P* = 0.0027; *N* = 3 per group; Fig. 2e). PolyI:C exposure in WT neurospheres also increased the in vitro expression of *Tlr3* (~1.5-fold), *RelA* (~2.0-fold) and *Sp1* (~1.6-fold) in comparison to WT saline group (Student’s two-tailed *t*-test; *N* = 3 per group; Fig. 2e). In contrast to WT, polyI:C administration to Sdy neurospheres did not increase *Tlr3* expression, and significantly reduced *RelA* (~0.5-fold) and *Sp1* (~0.6-fold) expression compared to the Sdy saline group as analyzed using Student’s two-tailed *t*-test (*N* = 3 per group; Fig. 2f). Overall, these results confirmed our in vivo data that polyI:C increases dysbindin-1 expression and that dysbindin-1 is necessary for polyI:C-induced increases in *Tlr3-RelA*-*Sp1* expression. They also indicated that polyI:C and dysbindin-1 have direct and rapid effects in SVZ cells.

In order to confirm that *Tlr3*, *RelA* and *Sp1* were specifically regulated by dysbindin-1, we carried out a rescue experiment. Murine *Dtnbp1* plasmid was nucleofected into neurospheres from Sdy mice (Fig. 2g). Twenty-four hours post nucleofection, neurospheres were incubated with saline or polyI:C for 3 h. *Dtnbp1* nucleofection resulted in detectable *Dtnbp1* mRNA expression in saline exposed neurospheres (Fig. 2h). PolyI:C caused a slight increase in *Dtnbp1* mRNA expression compared to saline incubated neurospheres (Fig. 2h), suggesting Sdy neurospheres nucleofected with *Dtnbp1* were also responsive to polyI:C. Compared to mock-nucleofected Sdy neurospheres, we found a significant main effect of rescuing *Dtnbp1* mRNA expression on *Tlr3* expression (~4.0-fold) increase regardless of treatment (two-way ANOVA; *F*(1,8) = 54.11; *P* < 0.0001; *N* = 3 per group). Similarly, we found a significant main effect of *Dtnbp1* nucleofection to Sdy neurospheres on *RelA* (*F*(1,8) = 11.33; *P* = 0.0098) and *Sp1* (*F*(1,8) = 9.031; *P* = 0.0169) mRNA expression as determined with two-way ANOVA (*N* = 3 per group; Fig. 2h). Together, our data show that dysbindin-1 intrinsically regulated the expression of *Tlr3*, *RelA* and *Sp1* in SVZ cells.

### SVZ microglia and leucocytes are altered by dysbindin-1 gene and polyI:C

**Fig. 3:**
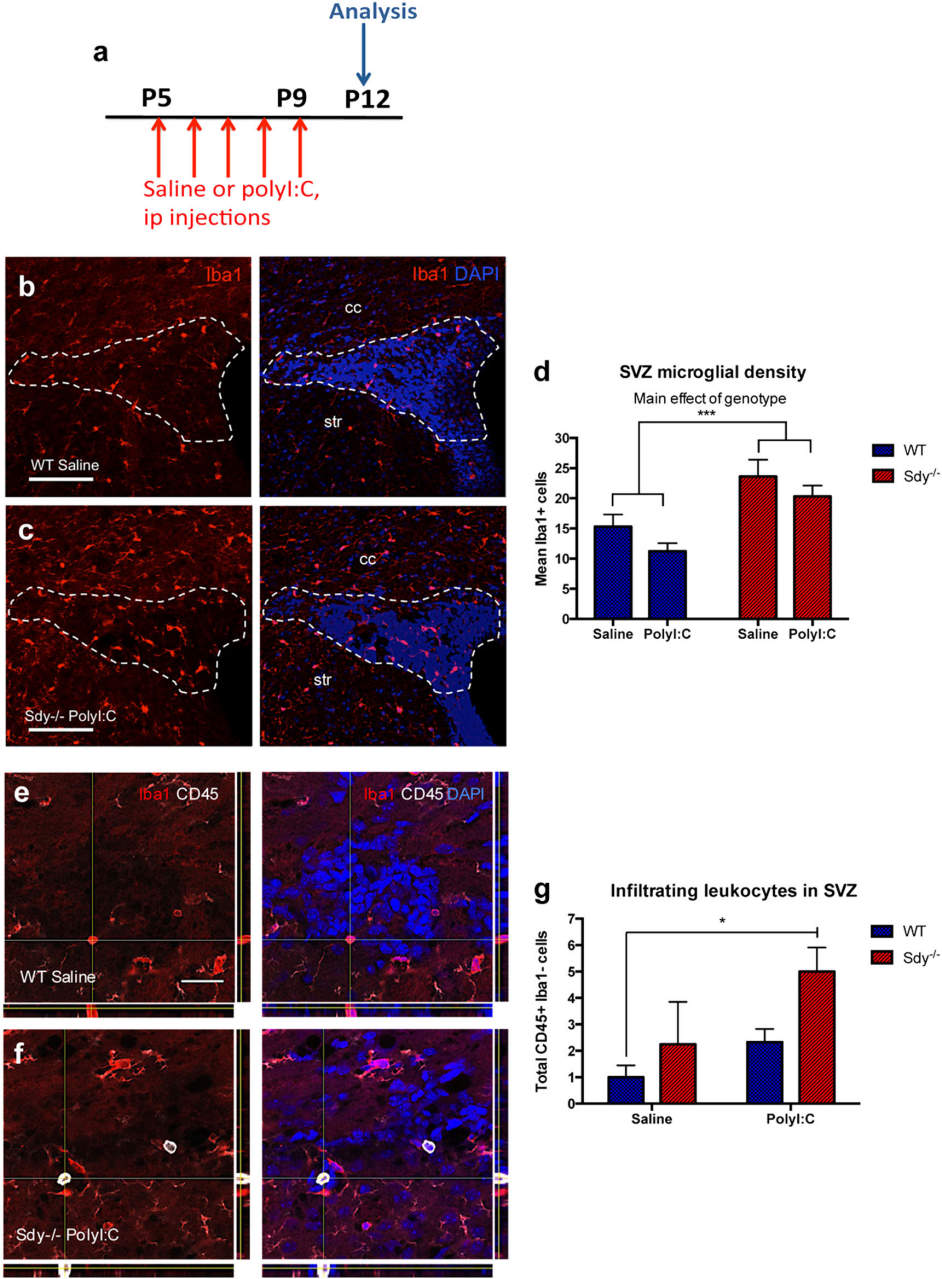
The SVZ of Sdy mice contain more microglia and exhibit increased leucocyte infiltration following peripheral polyI:C administration. **a** Experimental design showing the time-points of postnatal saline or polyI:C injections in WT and Sdy mice, SVZ samples were collected for analysis at P12. **b**, **c** Immunohistochemistry for Iba1 revealed a higher density of immunopositive cells in the SVZ (outlined), corpus callosum (cc) and striatum (str) of Sdy polyI:C compared to control WT saline group. **d** Sdy mice at P12 had significantly more Iba1+ cells in the SVZ regardless of treatment. **e**, **f** Double immunolabelling for Iba1 and CD45 in WT saline and Sdy polyI:C mice. Orthogonal views show a CD45-/Iba1+ cell in the SVZ of a WT saline mouse and a CD45+/Iba1− cell in the SVZ of a Sdy polyI:C mouse. **g** The total number of CD45+/Iba1− cells in the SVZ at P12 was increased in Sdy polyI:C compared to WT saline mice. All values are mean ± s.e.m. of *N* = 4 per WT groups and *N* = 6 per Sdy groups. Data were analyzed using two-way ANOVA for (**d**) with Tukey’s test for (**g**). *P < 0.05 and ***P < 0.001. Scale bars 100 μm for (**b**, **c**) and 25 μm for (**e**, **f**)

We next investigated polyI:C and dysbindin-1 loss on immune cells in the SVZ. We studied Iba1+ cells in the SVZ at P12 following P5-9 saline or polyI:C injections in WT (*N* = 4) and Sdy (*N* = 6) mice (Fig. 3a). Although many immune cells can express Iba1 in the periphery, in resting conditions Iba1 is a commonly used marker for microglia in the brain.^28^ Qualitative examination revealed Sdy mice contained more Iba1 + SVZ cells compared to WT mice (Fig. 3b, c). Sdy mice also exhibited more Iba1+ cells in the corpus callosum and striatum than WT mice (Fig. 3b, c). Two-way ANOVA revealed a main effect of the genotype on SVZ Iba1+ cell numbers (*F*(1,16) = 18.93; *P* = 0.0005) regardless of treatment (Fig. 3d). However, polyI:C compared to saline injections did not increase SVZ Iba1+ cell numbers or Iba1+ immunofluorescence in WT or Sdy mice (Fig. 3b-d).

Systemic polyI:C injections activate leucocytes which can enter the brain via the SVZ. We quantified cells with high CD45 expression, total absence of Iba1 immunoreactivity and amoeboid morphology (Fig. 3e, f), which were likely infiltrating leucocytes. We found a significant main effect of the polyI:C treatment (*F*(1,16) = 5.919; *P* = 0.0271) and a main effect of the genotype (*F*(1,16) = 5.446; *P* = 0.0330) on the total number of CD45+/Iba1− cells in the postnatal SVZ (two-way ANOVA; *N* = 4–6 per group; Fig. 3g). Tukey’s post hoc test showed that polyI:C injections significantly increased CD45+/Iba1− cell numbers in the postnatal Sdy SVZ compared to WT saline group (*P* = 0.0184; Fig. 3g). However, polyI:C injections in WT pups resulted in non-significant leucocyte increases compared to WT saline (two-way ANOVA with Tukey’s test; Fig. 3g). In summary, loss of dysbindin-1 increased postnatal SVZ microglial numbers, and peripheral polyI:C in Sdy mice increased SVZ leucocyte infiltration.

### Abnormal SVZ neurodevelopment in Sdy mice given postnatal polyI:C injections

**Fig. 4:**
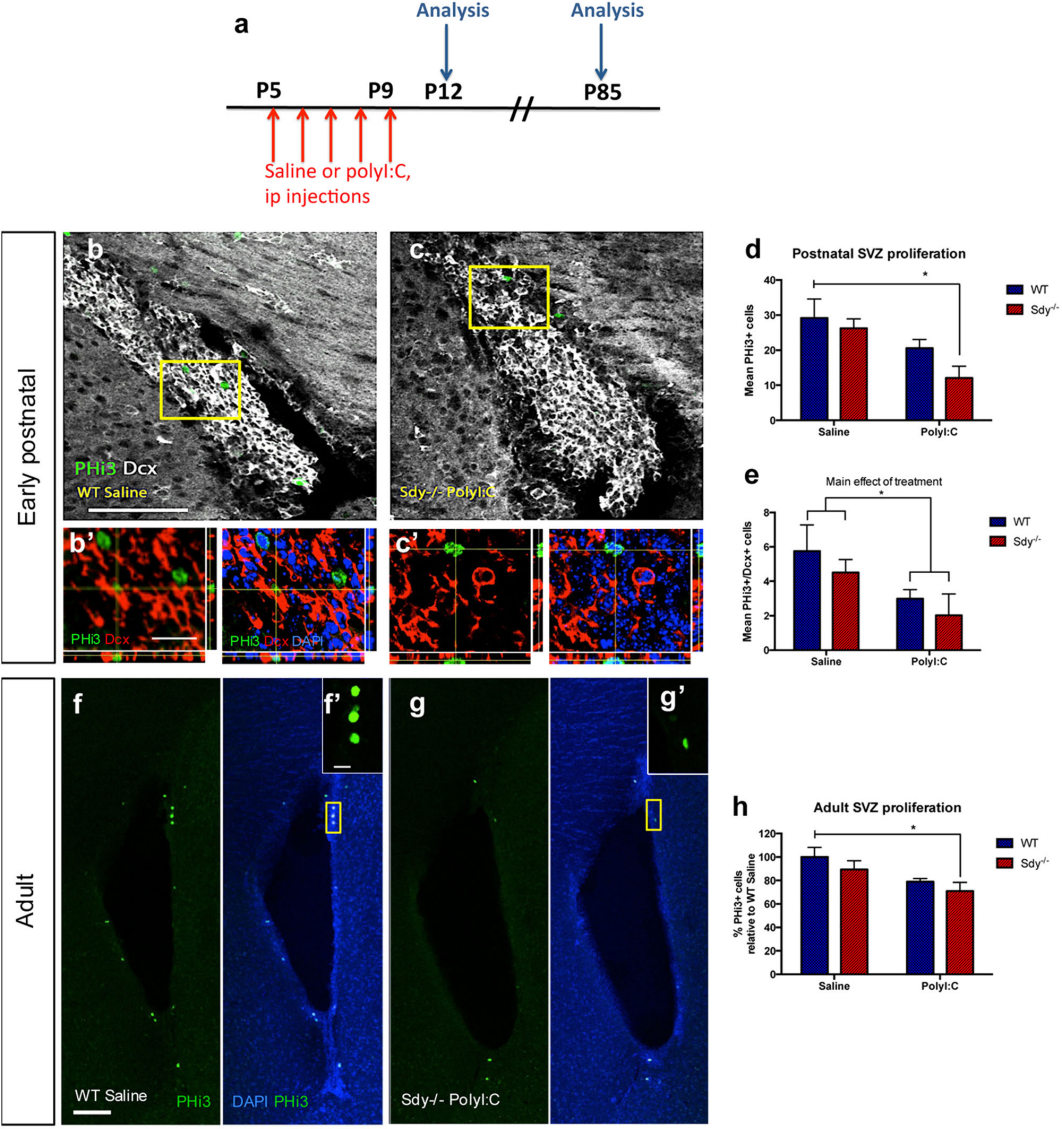
Reduced postnatal and adult SVZ proliferation in Sdy polyI:C mice. **a** Postnatal WT and Sdy mice were injected with saline or polyI:C from P5-9 and brains were collected for analysis at postnatal age P12 or adult P85. **b**, **c** Representative images of double-labelled PHi3+/Dcx+ mitotic neuroblasts in the SVZ of WT saline group compared to Sdy polyI:C group. **b**’,**c**’ High-magnification confocal z stacks showing boxed areas in **b** and **c** with orthogonal views of a PHi3+/Dcx+ cell in **b**’ and a PHi3+/Dcx− cell in **c**’. **d** The mean number of PHi3+ cells was significantly reduced in Sdy polyI:C group in comparison to the WT saline group. **e** PolyI:C treatment had a significant main effect on the number of PHi3+/Dcx+ mitotic neuroblasts in the SVZ at P12. **f**, **g** Representative images showing PHi3+ cells in the SVZ of adult WT saline and Sdy polyI:C mice, with higher magnification of yellow-boxed areas illustrated in insets. **h** The number of proliferative (PHi3+) cells in the adult SVZ of Sdy polyI:C mice was significantly decreased compared to WT saline mice. All values are mean ± s.e.m. of *N* = 3 per group for (**d**, **e**) and *N* = 6 per group for (**h**). Data were analyzed using two-way ANOVA for (**e**) with Tukey’s post hoc test for (**d**, **h**). *P < 0.05. Scale bars 100 μm for (**b**, **c** and **f**, **g**), 20 μm for (**b**’, **c**’) and 15 μm for (**f**’, **g**’)

We next investigated effects of polyI:C and dysbindin-1 loss on SVZ neurodevelopment at P12 and P85 (Fig. 4a). Figure 4b shows mitotic phosphohistone-3+(PHi3) SVZ cells with co-immunolabeling of the neuroblast marker doublecortin (Dcx) in a WT saline mouse. There were fewer PHi3+/Dcx+ cells in the postnatal SVZ of the Sdy polyI:C group in comparison to WT saline group (Fig. 4b, c). Although two-way ANOVA showed only a significant main effect of polyI:C treatment on the total number of SVZ PHi3+ cells (*F*(1,8) = 9.65; *P* = 0.0145; *N* = 3 per group), Tukey’s post hoc test showed that PHi3+ cell numbers were significantly reduced in Sdy polyI:C group compared to the WT saline group (Fig. 4d). In addition, there was a significant main effect of polyI:C injections on the number of PHi3+/Dcx+ cells in the P12 SVZ (two-way ANOVA; *F*(1,8) = 5.763; *P* = 0.0431; *N* = 3 per group; Fig. 4e), however Tukey’s test did not reveal a specific difference among the different groups.

Most schizophrenia symptoms arise in adulthood even though genetic and environmental risk factors may affect perinatal brain development. We therefore asked if the Sdy x polyI:C postnatal effect might persist (Fig. 4f–h). Two-way ANOVA revealed a significant main effect of postnatal polyI:C treatment on the number of PHi3+ cells in adult SVZ (*F*(1,20) = 8.449; *P* = 0.0087; *N* = 6 per group). Tukey’s post hoc test showed that the number of PHi3+ cells was specifically reduced in adult Sdy polyI:C group compared to the WT saline group (*P* = 0.031; Fig. 4f–h), indicating a long-lasting impact.

**Fig. 5:**
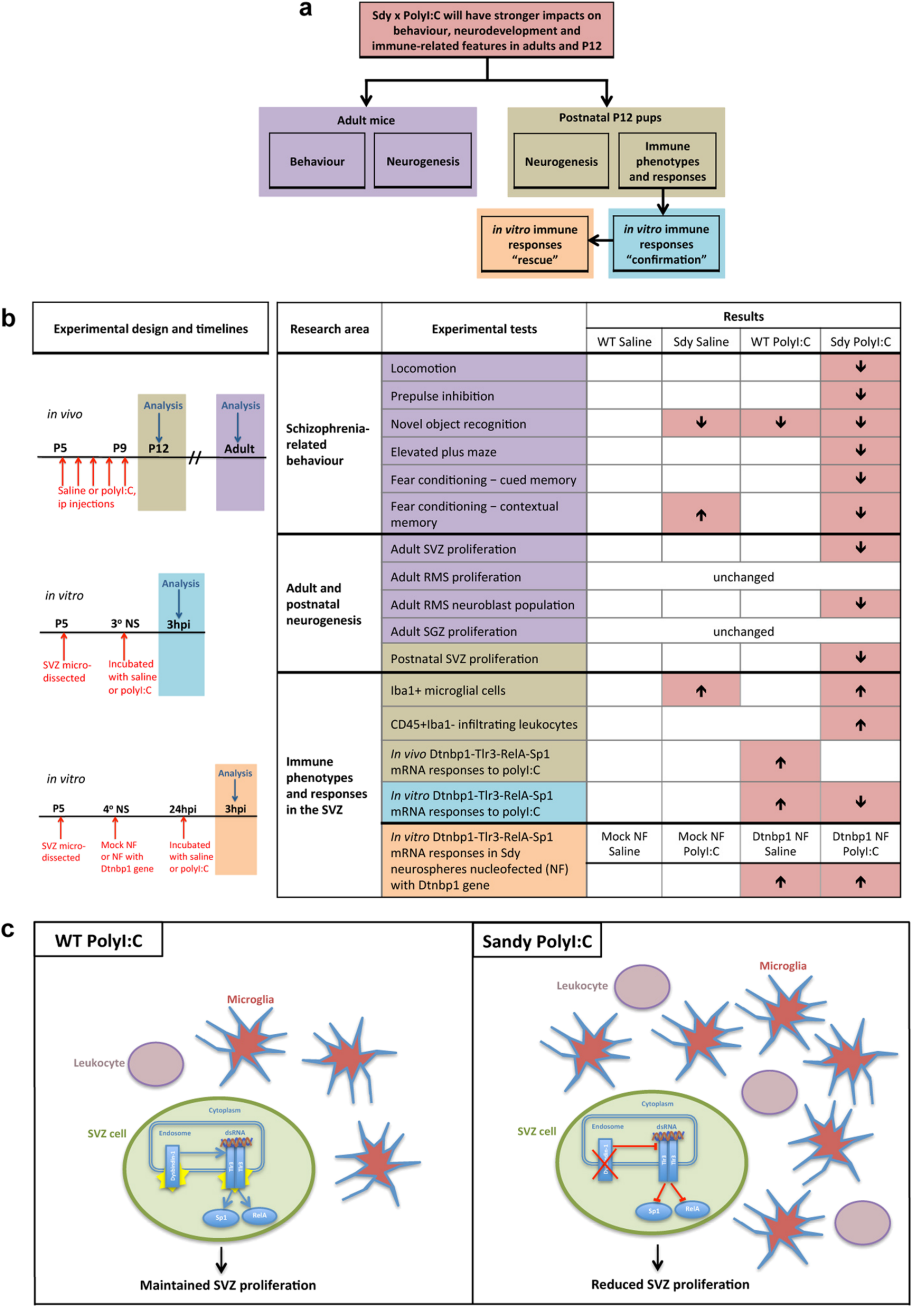
Summary of the main study hypothesis, experimental design and results. **a** Our major hypothesis. **b** Study design, research areas, experimental tests and outcomes of our study. Sdy polyI:C mice had more effects than the Sdy or PolyI:C alone groups. Note, the experimental design and timelines are colour-coded and coordinated with the experimental tests in (**b**) and overall hypothesis in (**a**). Also note *Dtnbp1* is not expressed in Sdy samples. **c** Schematic diagram illustrating the main molecular and cellular results of the study. PolyI:C induced dysbindin-1 (*Dtnbp1*) expression, and *Dtnbp1* enhanced *Tlr3* expression in SVZ cells. Sandy SVZ cells that lack dysbindin-1 showed blocked *Tlr3*-*RelA*-*Sp1* immune response to polyI:C. Microglial number was higher in postnatal Sandy SVZ than in WT controls. Leucocyte permeability was increased in postnatal SVZ of Sandy, but not WT mice given systemic polyI:C injections. Postnatal polyI:C injections reduced SVZ proliferation in postnatal Sandy mice, which continued to adulthood

We also characterized the neuroblast population and proliferation in the destination of SVZ cells, the rostral migratory stream (RMS) at P85 (Supplementary Figure 3a). The neuroblast population was decreased in adult Sdy mice given postnatal polyI:C (Supplementary Figure 3b-c). Although two-way ANOVA showed a significant main effect of polyI:C treatment on the RMS neuroblast population (*F*(1,8) = 32.32; *P* = 0.0005; *N* = 3 per group), Tukey’s test revealed that it was only significantly reduced in the Sdy polyI:C group in comparison to WT saline and Sdy saline groups (*P* < 0.01; Supplementary Figure 3d). The number of PHi3+ cells in the RMS was not significantly different among the four groups (Supplementary Figure 3e). Our results indicate the genetic or environmental risk factors alone were not sufficient to affect SVZ neurodevelopment. However a combination of these factors caused both a short- and long-term impact on SVZ neurodevelopment. We present a schematic summary of our main results in Fig. 5.

We next asked whether proliferation is affected in the dentate gyrus subgranular zone (SGZ), the other major neurogenic niche of the adult brain. We found no statistically significant difference in the numbers of SGZ cells positive for the proliferation marker Ki67+ or double positive for Ki67+/Dcx+ (Supplementary Figure 4a-c). These results suggest the SVZ niche is especially vulnerable to this model of GxE interaction.

## Discussion

We show that the schizophrenia-relevant dysbindin-1 (*Dtnbp1*) gene is necessary for the innate immune response to the viral mimetic polyI:C, and that polyI:C in turn increases dysbindin-1 expression. We also measured cellular and behavioural parameters linked to schizophrenia and found that several of these parameters were only significantly disrupted in the combined Sdy polyI:C mice. Hence, our findings suggest insights into the underlying mechanisms that lead to synergistic effects of distinct classes of risk factors for schizophrenia.

In order to confirm the relevance of the Sdy polyI:C mice to schizophrenia, we carried out several behavioural tests germane to the disease and to other neuropsychiatric disorders. Adult Sdy mice, given polyI:C postnatally, had reduced PPI, suggesting sensorimotor gating deficits similar to what has been observed in schizophrenia. Although adult Sdy mice reportedly have decreased PPI,^29^ this did not reach statistical significance by itself in our study. We found Sdy polyI:C mice had deficits in object recognition memory, an endophenotype suggestive of abnormal cognitive function which can be observed in schizophrenia as well as depression.^30,31^ WT polyI:C and Sdy saline mice also had disrupted object recognition in adulthood, supporting previous reports.^24,32,33^ Also, the Sdy polyI:C group showed impaired fear memories to both contextual and tone cues, indicating attenuated memory of fearful events. The reduced locomotion in our Sdy polyI:C mice might indicate disrupted neural function since novel environments normally lead to increased dopamine release and locomotor activity in rodents.^34^ PolyI:C injections in Sdy mice enhanced anxiety-like behaviour as they spent significantly less time in the open compared to closed arm. This supports a previous report in which human *DTNBP1* variation has been associated with anxiety in addition to schizophrenia^35^ Taken together, dysbindin-1 mutation in combination with postnatal immune activation increased the susceptibility to developing some schizophrenia-like behavioural symptoms. The similarities of some of these behaviours with those disrupted in schizophrenia give our model good face validity for the positive symptoms of the disease.

We then found an interesting impact of dysbindin-1 mutation on the immune response to polyI:C. *Tlr3*, *RelA* and *Sp1* mRNA expression levels increased in the SVZ of WT mice given polyI:C, demonstrating a normal immune response to polyI:C in a neurogenic niche. Unexpectedly, dysbindin-1 expression also increased in response to polyI:C. We do not know how this occurs, however there is evidence that RelA and Sp1 might regulate dysbindin-1 expression.^36,37^ In contrast to WT mice, Sdy mice did not exhibit increased *Tlr3*, *RelA* and *Sp1* following polyI:C administration, suggesting that dysbindin-1 is necessary for this canonical immune signalling response. This is consistent with another study showing impaired TLR3 expression following polyI:C administration to monocytes derived from schizophrenic patients.^38^

Importantly, we further supported these findings through a nucleofection experiment in which *Dtnbp1* gene rescued *Tlr3*, *RelA* and *Sp1* expression in Sdy neurospheres. This might indicate that *Dtnbp1* has a direct functional effect on *Tlr3* followed by *RelA* and *Sp1* induction. As well, the comparable increase in *Tlr3*-*RelA-Sp1* expression in *Dtnbp1* nucleofected neurospheres with or without polyI:C might be due to a dosage effect; *Dtnbp1* overexpression may have caused increased *Tlr3*-*RelA-Sp1* to reach a threshold that polyI:C treatment could not surpass. In comparison to non-nucleofected Sdy neurospheres, polyI:C treatment to mock-nucleofected Sdy neurospheres showed a masked reduction in *RelA* and *Sp1* mRNA which might be due to the nucleofection protocol itself that slightly altered the response of neurospheres. Future in vivo rescue experiments would further support our in vitro results. Despite these caveats, together our results imply that dysbindin-1 is functionally required to induce the innate immune response to viral-like infection in the SVZ neurogenic niche.

The microglial population in the postnatal SVZ of Sdy mice was increased, supporting the role of dysbindin-1 in regulating immune function. These results are interestingly similar to another study that showed increased microglial density in postmortem schizophrenic brains,^39^ and may also suggest a developmental origin of microglial dysregulation in schizophrenia. In addition, leucocytes infiltrating into the SVZ were investigated in our study since peripheral polyI:C could stimulate immune cell recruitment into the brain. The number of leucocytes found in the SVZ of postnatal WT mice was not altered by polyI:C suggesting the blood-brain barrier remained unperturbed.^40,41^ However, we found increased number of leucocytes in the postnatal SVZ of the Sdy polyI:C mice, and this supports a previous study reporting increased lymphocyte infiltration in the hippocampus of schizophrenic patients.^42^ We used Iba1 to mark brain microglia^28^ and CD45+/Iba1− immunoreactivity to label peripheral leucocytes. No current antibody-based marker perfectly distinguishes microglia from macrophages or other peripheral immune cells, although TMEM119 has been proposed as specific for microglia and could help categorize these populations in future studies.^43^ As well, the plasticity of monocytes and macrophages is well known.^44–46^ Ultimately, adoptive transfer would be needed for definitive attribution of the peripheral source of immune cells, however that was beyond the scope of this work. This limitation notwithstanding, we provide evidence for the first time to our knowledge that dysbindin-1 regulates immune cell homeostasis in the developing SVZ.

We expected polyI:C injections to increase SVZ microglial numbers in WT mice. This was not observed, showing that systemic injections were insufficient for increasing microglial numbers even though *Tlr3* expression indicated inflammation at the molecular level. The increased numbers of microglia in Sdy SVZs may also seem paradoxical since Sdy mice had blocked *Tlr3*-*RelA*-*Sp1* responses to polyI:C suggesting a dampened inflammatory response. However, the increased microglial number does not necessarily imply microglial activation. We evaluated the expression of *Tlr3*-*RelA*-*Sp1* genes in all SVZ cells, which include microglia,^27^ and the role of these genes in microglial proliferation remains unclear. Therefore, the increase in microglial number in Sdy mice may indicate that the dysbindin-1 mutation itself (but not polyI:C) contributed to this phenotype during early development. In fact, dysbindin-1 is strongly expressed during late embryonic development (E14/E18),^47^ which coincides with massive microglial proliferation at E16.^48^ Thus, dysbindin-1 may limit the embryonic microglial pool, and dysbindin-1 loss may expand the prenatal microglial population resulting in increased postnatal numbers.

In addition to immune regulation, we showed that dysbindin-1 has a neurodevelopmental function in the SVZ in the context of polyI:C-induced inflammation. We found evidence for reduced proliferation in both postnatal and adult Sdy mice given early postnatal polyI:C injections, indicating a role for dysbindin-1 in maintaining proliferation following inflammation. Although there is no direct evidence that *Tlr3* and *Sp1* regulate SVZ proliferation, *RelA* is directly involved in this function in adult SVZ neurospheres.^49^ Interestingly, increased physical activity was previously shown to associate with increased SVZ proliferation.^50^ We found that postnatal polyI:C injections to Sdy but not WT mice resulted in reduced locomotor activity which may have contributed to the decreased SVZ proliferation. Similarly, neuroblast populations in the RMS of the Sdy polyI:C mice were reduced which may be explained by lower rates of neuroblast migration from the SVZ. This is further supported since SVZ proliferation was diminished in the Sdy polyI:C mice, reducing the pool of neuroblasts available for RMS migration. We believe these results are relevant since neurons born in the human SVZ are thought to migrate to the olfactory bulbs and cerebral cortex postnatally^51^ and to the striatum^52^ in adults. These regions are likely involved in multiple symptoms in schizophrenia.^53^ Interestingly, previous work led to the hypothesis that GxE interactions converge to regulate dopamine release and neurogenesis in the ventral striatum, particularly in the Islands of Calleja, in which neuronal clusters generated from the SVZ are much expanded in humans.^54^ Genetic alteration in the dopamine D2 receptor is a major risk factor for developing schizophrenia^55^ and the dopamine D3 receptor is necessary for SVZ neurogenesis.^56^ Thus it would be important in future studies to examine striatal dopamine release and function using the Sdy polyI:C mice. Together, we have shown that dysbindin-1 maintained certain features of normal neurodevelopment following perinatal viral-like infection. These findings expand our knowledge of the classical functions of dysbindin-1 in neurotransmitter release and receptor internalization,^3,7^ and show for the first time a neurodevelopmental function for dysbindin-1 in the SVZ neurogenic niche.

Postmortem sections have revealed evidence for decreased hippocampal dentate gyrus neurogenesis in humans with schizophrenia.^57^ The hippocampus encodes memories and since the Sdy polyI:C group had disrupted fear memories to both contextual and tone cues and also exhibited deficits in object recognition memory, we hypothesized that hippocampal neurogenesis may be altered. However, there were no statistically significant differences from controls in adult SGZ proliferation after polyI:C treatment in both WT and Sdy mice. Other experiments also showed that Sdy mice do not have significantly altered SGZ proliferation but revealed altered differentiation rates of newborn neurons.^58,59^ In future work it will be important to further probe the combination of polyI:C and dysbindin-1’s potential role in regulating different aspects of the neurogenic lineage in the hippocampal neurogenic niche.

In conclusion, we report novel evidence that dysbindin-1 is functionally required for initiating a normal immune response to an environmental stimulus, polyI:C, and that it is necessary for normal neurodevelopment, immune homeostasis and behavioural functions. Many groups studying schizophrenia stimulate the immune system of pregnant dams, therefore it will be interesting to see if such prenatal polyI:C stimulation in Sdy mice also causes effects similar to this study. This is especially important since recent genome-wide association studies (GWAS) support a strong association between schizophrenia and immunity.^55,60^ Our work provides molecular insight into how a genetic defect related to schizophrenia in combination with an environmental insult can result in amplified abnormalities relevant to the disease. It will be interesting for future studies to use cell cultures to determine if humans with schizophrenia and dysbindin-1 (*DTNBP1*) mutations have altered homoeostatic or inflammation-induced immune responses. Also, it will be fascinating to discover if other schizophrenia risk genes may be functionally required for regulating immune function in response to environmental risk factors.

## Methods

### Animals

Dysbindin-1 mutant (Sandy-Sdy) mice on C57BL/6 background were obtained from the Jackson Laboratory in Canada by our co-author Professor Lalit Srivastava (McGill University). Animal use was in accordance with the guidelines of the Canadian Council of Animal Care and was approved by the McGill University Animal Care Committee.

All animal procedures were carried out with Oxford University Research Ethics Committee approval in accordance with the Animals (Scientific Procedures) Act of 1986 (UK).

### PolyI:C administration

5 mg/kg polyI:C (Invivogen tlrl-pic, 1 mg/ml stock solution in 0.9% NaCl) or equivalent volumes of normal saline (0.9% NaCl, Aqupharm 01300101) were injected intraperitoneally (i.p.) into postnatal Sdy and WT pups from P5 to P9 (five injections, once daily). In all experiments, animals within the same litter were divided into two groups receiving either saline or polyI:C injections in order to avoid effects of litter variations.

### Perfusion and tissue preparation

Postnatal day 12 and adult mice were deeply anaesthetized with an overdose of 0.05 ml or 0.1 ml, respectively, i.p. injection of pentobarbitone (200 mg). They were then transcardially perfused with ice-cold 0.9% normal saline followed by ice-cold 4% paraformaldehyde (PFA) in phosphate buffered saline (PBS). Brains were carefully dissected out and post-fixed for 24 h in 4% PFA at 4 °C followed by cryoprotection for 3–4 days in 30% sucrose in phosphate buffer (PB) at 4 °C. Brains were snap-frozen on dry ice and stored at −80 °C.

### Microtome sectioning

Sliding microtome (Leica SM2000R) was used to cut frozen brains into 30 μm coronal sections. Frozen brains were first mounted onto a frozen stage with 0.1 M PB (pH 7.2) then serially cut and placed as free-floating sections in cryoprotectant in 48-well plates and stored at −20 °C. The mouse brain stereotaxic atlas by Franklin & Paxinos (2001) was used as a reference for determining the coronal coordinates of the brain regions.^61^ The sections collected from the RMS are 2.46 to 1.94 mm relative to bregma; whereas, the SVZ are 1.10 to −0.82 mm to bregma and SGZ are −0.94 mm to −2.70 mm to bregma.

### Neurosphere culture

Postnatal day 5 littermates were anaesthetized by hypothermia followed by decapitation and whole brains were dissected out and sectioned into 0.5 mm coronal slices using a neonatal mouse brain slicer matrix (Zivic instruments BSMNS005-1). Under a sterile laminar flow hood, both striatal and septal SVZ were microdissected in ice-cold Advanced DMEM (Dulbecco’s Modified Eagle Medium, Gibco) and were pooled together from *N* = 2–5 littermates per group. SVZ tissues were briefly centrifuged; supernatant aspirated and tissues were dissociated into single cells by incubation in 1 ml Accutase solution (Sigma A6964). Then two washes in neurobasal A medium (Gibco 10888-022) containing B27 (Gibco 17504), Glutamax (Gibco 35050-038), Penicillin/Streptomycin (Gibco 15070-063) before resuspending the cell pellets in the medium with added growth factors of 20 ng/ml EGF (Sigma E4127) and 20 ng/ml bFGF (R&D Systems 3139-FB-025). Cells were counted using a haemocytometer, plated at a density of 100,000 cells/ml in six-well plates (2 ml/well) and incubated at 37 °C and 5% CO_2_. To avoid neurosphere adhesion to the wells, the six-well-plates were coated with 1.2 mg/ml poly-HEMA (poly(2-hydroxyethyl methacrylate), Sigma P3932) in 95% ethanol and were allowed to air dry inside sterile laminar hood before use. Neurospheres were passaged and given fresh media every fourth day.

### Nucleofection

A total of 1 × 10^6^ dissociated cells from quaternary passaged Sdy neurospheres were nucleofected with pCMV6-Dtnbp1 expression plasmid (OriGene MR205351) or mock-nucleofected according to the protocol of Nucleofector Kit for Mouse Neural Stem Cells (LONZA VPG-1004). Nucleofected cells were immediately transferred into one-well in poly-HEMA coated 12-well plates that contained culture medium and incubated at 37 °C and 5% CO_2_. After 24 h, cells were suspended in fresh culture medium and each sample was split into two wells (0.5 ml/well) in poly-HEMA coated 24-well plates and equal volumes of pre-warmed saline or polyI:C (50 μg/ml) were added. This experimental design worked as “within wells control” and therefore any detected response is likely due to the treatment itself and not due to variation in cell populations. Neurospheres were incubated at 37 °C and 5% CO_2_ for 3 h before use in RT-qPCR.

### Statistics

Data were analyzed using GraphPad Prism (version 7.0a). Student’s *t*-test was used to analyze differences between two groups. Two or three-way analysis of variance (ANOVA) was used to analyze two or more groups with two or three independent variables, respectively, and was followed by Tukey’s multiple comparisons test where appropriate. Data were presented as mean ± standard error of the mean (s.e.m).

### Data availability

All relevant data will be made available upon request.

## Electronic supplementary material

Supplementary Information

## References

[1] Ayhan Y, McFarland R, Pletnikov MV (2016). Animal models of gene-environment interaction in schizophrenia: A dimensional perspective. Prog. Neurobiol..

[2] Straub RE (2002). Genetic variation in the 6p22.3 gene DTNBP1, the human ortholog of the mouse dysbindin gene, is associated with schizophrenia. Am. J. Hum. Genet..

[3] Talbot, K. et al. in *Handbook of Neurochemistry and Molecular Neurobiology: Schizophrenia*, 3rd edn, Springer, Boston, MA 107–241 (2009).

[4] Weickert CS, Rothmond DA, Hyde TM, Kleinman JE, Straub RE (2008). Reduced DTNBP1 (dysbindin-1) mRNA in the hippocampal formation of schizophrenia patients. Schizophr. Res..

[5] Weickert CS (2004). Human dysbindin (DTNBP1) gene expression in normal brain and in schizophrenic prefrontal cortex and midbrain. Arch. Gen. Psychiatry.

[6] Tang J (2009). Dysbindin-1 in dorsolateral prefrontal cortex of schizophrenia cases is reduced in an isoform-specific manner unrelated to dysbindin-1 mRNA expression. Hum. Mol. Genet.

[7] Talbot K (2004). Dysbindin-1 is reduced in intrinsic, glutamatergic terminals of the hippocampal formation in schizophrenia. J. Clin. Invest..

[8] Brown AS (2011). The environment and susceptibility to schizophrenia. Prog. Neurobiol..

[9] Meyer U, Schwendener S, Feldon J, Yee BK (2006). Prenatal and postnatal maternal contributions in the infection model of schizophrenia. Exp. Brain Res..

[10] Piontkewitz Y, Arad M, Weiner I (2012). Tracing the development of psychosis and its prevention: what can be learned from animal models. Neuropharmacology.

[11] Alexopoulou L, Holt AC, Medzhitov R, Flavell RA (2001). Recognition of double-stranded RNA and activation of NF-kappaB by toll-like receptor 3. Nature.

[12] Doyle SL (2013). Nuclear factor kappaB2 p52 protein has a role in antiviral immunity through IkappaB kinase epsilon-dependent induction of Sp1 protein and interleukin 15. J. Biol. Chem..

[13] Gu L, Findley HW, Zhou M (2002). MDM2 induces NF-kappaB/p65 expression transcriptionally through Sp1-binding sites: a novel, p53-independent role of MDM2 in doxorubicin resistance in acute lymphoblastic leukemia. Blood.

[14] Perkins ND, Agranoff AB, Pascal E, Nabel GJ (1994). An interaction between the DNA-binding domains of RelA (p65) and Sp1 mediates human immunodeficiency virus gene activation. Mol. Cell Biol..

[15] Yurochko AD, Mayo MW, Poma EE, Baldwin AS, Huang ES (1997). Induction of the transcription factor Sp1 during human cytomegalovirus infection mediates upregulation of the p65 and p105/p50 NF-kappaB promoters. J. Virol..

[16] Song XQ, Lv LX, Li WQ, Hao YH, Zhao JP (2009). The interaction of nuclear factor-kappa B and cytokines is associated with schizophrenia. Biol. Psychiatry.

[17] Ben-Shachar D, Karry R (2007). Sp1 expression is disrupted in schizophrenia; a possible mechanism for the abnormal expression of mitochondrial complex I genes, NDUFV1 and NDUFV2. PLoS ONE.

[18] Pinacho R (2014). Increased SP4 and SP1 transcription factor expression in the postmortem hippocampus of chronic schizophrenia. J. Psychiatry Res..

[19] Workman AD, Charvet CJ, Clancy B, Darlington RB, Finlay BL (2013). Modeling transformations of neurodevelopmental sequences across mammalian species. J. Neurosci..

[20] Semple BD, Blomgren K, Gimlin K, Ferriero DM, Noble-Haeusslein LJ (2013). Brain development in rodents and humans: Identifying benchmarks of maturation and vulnerability to injury across species. Prog. Neurobiol..

[21] Blomstrom A (2016). Associations between maternal infection during pregnancy, childhood infections, and the risk of subsequent psychotic disorder--a Swedish Cohort Study of Nearly 2 Million Individuals. Schizophr. Bull..

[22] Clarke MC, Tanskanen A, Huttunen M, Whittaker JC, Cannon M (2009). Evidence for an interaction between familial liability and prenatal exposure to infection in the causation of schizophrenia. Am. J. Psychiatry.

[23] Ibi D (2010). Combined effect of neonatal immune activation and mutant DISC1 on phenotypic changes in adulthood. Behav. Brain Res..

[24] Bhardwaj SK (2009). Behavioral characterization of dysbindin-1 deficient sandy mice. Behav. Brain Res..

[25] Bhardwaj SK, Stojkovic K, Kiessling S, Srivastava LK, Cermakian N (2015). Constant light uncovers behavioral effects of a mutation in the schizophrenia risk gene Dtnbp1 in mice. Behav. Brain Res..

[26] Falcao AM (2012). The path from the choroid plexus to the subventricular zone: go with the flow!. Front. Cell Neurosci..

[27] Goings GE, Kozlowski DA, Szele FG (2006). Differential activation of microglia in neurogenic versus non-neurogenic regions of the forebrain. Glia.

[28] Ito D (1998). Microglia-specific localisation of a novel calcium binding protein, Iba1. Brain Res. Mol. Brain Res..

[29] Carlson GC (2011). Dysbindin-1 mutant mice implicate reduced fast-phasic inhibition as a final common disease mechanism in schizophrenia. Proc. Natl Acad. Sci. USA.

[30] Elvevag B, Goldberg TE (2000). Cognitive impairment in schizophrenia is the core of the disorder. Crit. Rev. Neurobiol..

[31] MacQueen GM, Memedovich KA (2017). Cognitive dysfunction in major depression and bipolar disorder: Assessment and treatment options. Psychiatry Clin. Neurosci..

[32] Ibi D (2009). Neonatal polyI:C treatment in mice results in schizophrenia-like behavioral and neurochemical abnormalities in adulthood. Neurosci. Res..

[33] Feng YQ (2008). Dysbindin deficiency in sandy mice causes reduction of snapin and displays behaviors related to schizophrenia. Schizophr. Res..

[34] Feenstra MG, Botterblom MH, van Uum JF (1995). Novelty-induced increase in dopamine release in the rat prefrontal cortex in vivo: inhibition by diazepam. Neurosci. Lett..

[35] Voisey J (2010). A polymorphism in the dysbindin gene (DTNBP1) associated with multiple psychiatric disorders including schizophrenia. Behav. Brain Funct..

[36] Fu C, Chen D, Chen R, Hu Q, Wang G (2015). The schizophrenia-related protein dysbindin-1A is degraded and facilitates NF-Kappa B activity in the nucleus. PLoS ONE.

[37] Liao HM, Chen CH (2004). Mutation analysis of the human dystrobrevin-binding protein 1 gene in schizophrenic patients. Schizophr. Res..

[38] Muller N (2012). Impaired monocyte activation in schizophrenia. Psychiatry Res..

[39] Radewicz K, Garey LJ, Gentleman SM, Reynolds R (2000). Increase in HLA-DR immunoreactive microglia in frontal and temporal cortex of chronic schizophrenics. J. Neuropathol. Exp. Neurol..

[40] Stolp HB, Dziegielewska KM, Ek CJ, Potter AM, Saunders NR (2005). Long-term changes in blood-brain barrier permeability and white matter following prolonged systemic inflammation in early development in the rat. Eur. J. Neurosci..

[41] Fernandez-Lopez D (2012). Blood-brain barrier permeability is increased after acute adult stroke but not neonatal stroke in the rat. J. Neurosci..

[42] Busse S (2012). Different distribution patterns of lymphocytes and microglia in the hippocampus of patients with residual versus paranoid schizophrenia: further evidence for disease course-related immune alterations?. Brain Behav. Immunol..

[43] Bennett ML (2016). New tools for studying microglia in the mouse and human CNS. Proc. Natl Acad. Sci. USA.

[44] Hume DA (2015). The many alternative faces of macrophage activation. Front. Immunol..

[45] Nilsson R (2006). Transcriptional network dynamics in macrophage activation. Genomics.

[46] Gautier EL (2012). Gene-expression profiles and transcriptional regulatory pathways that underlie the identity and diversity of mouse tissue macrophages. Nat. Immunol..

[47] Ghiani CA (2010). The dysbindin-containing complex (BLOC-1) in brain: developmental regulation, interaction with SNARE proteins and role in neurite outgrowth. Mol. Psychiatry.

[48] Alliot F, Godin I, Pessac B (1999). Microglia derive from progenitors, originating from the yolk sac, and which proliferate in the brain. Brain Res. Dev. Brain Res..

[49] Widera D, Mikenberg I, Elvers M, Kaltschmidt C, Kaltschmidt B (2006). Tumor necrosis factor alpha triggers proliferation of adult neural stem cells via IKK/NF-kappaB signaling. BMC Neurosci..

[50] Lee JC, Yau SY, Lee TM, Lau BW, So KF (2016). Voluntary wheel running reverses the decrease in subventricular zone neurogenesis caused by corticosterone. Cell Transplant.

[51] Paredes MF (2016). Extensive migration of young neurons into the infant human frontal lobe. Science.

[52] Ernst A (2014). Neurogenesis in the striatum of the adult human brain. Cell.

[53] Simpson EH, Kellendonk C, Kandel E (2010). A possible role for the striatum in the pathogenesis of the cognitive symptoms of schizophrenia. Neuron.

[54] Inta D, Meyer-Lindenberg A, Gass P (2011). Alterations in postnatal neurogenesis and dopamine dysregulation in schizophrenia: a hypothesis. Schizophr. Bull..

[55] Schizophrenia Working Group of the Psychiatric Genomics, C. (2014). Biological insights from 108 schizophrenia-associated genetic loci. Nature.

[56] Kim Y (2010). Dopamine stimulation of postnatal murine subventricular zone neurogenesis via the D3 receptor. J. Neurochem..

[57] Reif A (2006). Neural stem cell proliferation is decreased in schizophrenia, but not in depression. Mol. Psychiatry.

[58] Nihonmatsu-Kikuchi N (2010). Reduced rate of neural differentiation in the dentate gyrus of adult dysbindin null (sandy) mouse. PLoS ONE.

[59] Wang H (2014). Dysbindin-1C is required for the survival of hilar mossy cells and the maturation of adult newborn neurons in dentate gyrus. J. Biol. Chem..

[60] Network & Pathway Analysis Subgroup of Psychiatric Genomics, C. Psychiatric genome-wide association study analyses implicate neuronal, immune and histone pathways. *Nat. Neurosci.* **18**, 199–209 (2015).. 10.1038/nn.3922 PMC4378867 25599223

[61] Franklin, K. B. J. & Paxinos, G. *The Mouse Brain in Stereotaxic Coordinates*. (Academic Press, New York, 2001).

