# Supplementary material for: Schizophrenia-related dysbindin-1 gene is required for innate immune response and homeostasis in the developing subventricular zone
Source: NPJ Schizophr. 2018 Jul 23;4:15. doi: 10.1038/s41537-018-0057-5 (PMC6056426; doi:10.1038/s41537-018-0057-5)
Supplement: Supplementary file 1 — Supplementary Information [file 41537_2018_57_MOESM1_ESM.pdf]

SUPPLEMENTARY INFORMATION

SUPPLEMENTARY METHODS

Animals

The dysbindin-1 mutation that was originally identified on DBA/2J strain[62] was transferred to C57BL/6 background by the Jackson Laboratory through backcrossing Sdy mice with C57BL/6 mice for ten generations. All experiments used wild-type (WT) and dysbindin-1 mutant (Sandy-Sdy) mice on a C57BL/6 background. Adult mice used were direct progeny from heterozygotes, which were bred by the Srivastava laboratory at Douglas Mental Health University Institute. Heterozygous breeding pairs from the Srivastava laboratory (McGill University) were delivered to the Szele laboratory (University of Oxford) for experiments on pups, and the colony was maintained via HET x HET breeding. Behavioural analysis of adult animals was carried out in the Srivastava laboratory at the Douglas Mental Health University Institute. The same adult animals used in behavioural tests were later perfused and post-fixed and then shipped to the Szele laboratory (University of Oxford) for phenotypic analysis of adult brains. WT x WT and Sdy x Sdy breeding pairs (also direct progeny from heterozygotes) were set up in the University of Oxford in order to obtain sufficient number for experiments on postnatal pups. For behavioral tests and brain analysis at adulthood, only male mice were used. For postnatal analysis, males and females were used since it is difficult to distinguish the gender at a very young age. All mice were maintained in individually ventilated cages on 12-hour light/dark cycle with unlimited access to chow and water. Animal care in Montreal followed the guidelines of Canadian Council of Animal Care, and the procedures were approved by the McGill University Animal Care Committee. Procedures in Oxford were carried out with University of Oxford Research Ethics Committee approval, in accordance with the Animals (Scientific Procedures) Act of 1986 (UK) under Home Office PPL 30/3096. All efforts were

made to minimize animal suffering and distress.

Genotyping

Sdy mice harbour an autosomal recessive mutation in the *Dtnbp1* gene in chromosome 13 which arose spontaneously in the Jackson Laboratory in 1983.[62] The *Dtnbp1* mutation in Sdy mice is an in-frame deletion from intron 5 to intron 7, (hence deletion of exons 6 and 7) causing loss of amino acids 119-172, that results in dysbindin-1 protein degradation.[63]

The DNA samples from mouse earclips were extracted using E.Z.N.A Tissue DNA Kit (Omega Bio-tek D3396-02) according to the manufacturer protocol. For polymerase chain reaction (PCR) amplification, 4µl DNA sample was added to 21 µl reaction mixture. The primers for WT gene were as follows: forward-WT (50-TGAGCCATTAGGAGATAAGAGCA-30) and reverse-WT (50-AGCTCCACCTGCTGAACATT-30) yielding a PCR product of 472 base pairs (bp). The primers for Sdy gene were: forward-Sdy (50-TCCTTGCTTCGTTCTCTGCT-30) and reverse-Sdy (50-CTTGCCAGCCTTCGTATTGT-30) yielding a PCR product of 274bp. The reaction mixture was run in the following PCR program: 95°C for 5 min followed by 35 cycles of denaturation at 95°C for 20 sec, annealing at 56°C for 20 sec and elongation at 72°C for 30 sec, then a final elongation at 72°C for 5 min and the reaction was stopped at 4°C. The amplified PCR products were stored at -20°C until separated by gel electrophoresis.

Fluorescent immunohistochemistry

Free-floating sections were placed within porous well inserts (Sigma CLS3477) in 12-well plates and were washed in 0.1M PBS (pH7.4) followed by 15 min incubation in 50mM glycine in PBS to minimize auto-fluorescence. Sections were washed again in PBS before blocking for 1 hour in PBS+ (i.e. 10% donkey serum (Bio-Rad C06SBZ) and 0.1% Triton (Sigma X-100) diluted in PBS). Sections were then incubated in primary antibodies diluted

in PBS+ overnight at 4°C on a constant rocker. On the second day, sections were washed in PBS and incubated for 1 hour in Alexa conjugated secondary antibodies diluted 1:500 in PBS+. Sections were then washed in PBS, counterstained with DAPI (10µg/ml, MP Biomedicals 0215757450) before being rinsed in PB, mounted with FluorSave reagent (Calbiochem-Merck 345789) and coverslipped. No primary controls were performed in all experiments.

Antibodies

Rabbit anti-Phosophistone-3 (PHi3) 1:400 (Millipore 09-797) X donkey anti-rabbit Alexa Fluor 488 (Life Technologies A21206); goat anti-Doublecortin (Dcx) 1:100 (Santa-Cruz sc-8066) X donkey anti-goat Alexa Fluor 568 (Life Technologies A11057); goat anti-Iba1 1:200 (Abcam ab5076) X donkey anti-goat Alexa Fluor 568 (Life Technologies A11057); rat anti-CD45 1:200 (Millipore 05-1416, clone IBL5/25) X donkey anti-rat Alexa Fluor 647 (Abcam ab150155); rabbit anti-Tlr3 1:400 (Abcam ab62566) X donkey anti-rabbit Alexa Fluor 568 (Life Technologies A10042); rabbit anti-Ki67 1:200 (Abcam ab15580) X donkey anti-rabbit Alexa Fluor 568 (Invitrogen A10042); goat anti-Dcx 1:100 (Santa-Cruz sc8066) X donkey anti-goat Alexa Fluor 488 (Invitrogen A11055). We used a 1:500 dilution for all secondary antibodies.

Microscopy and quantification

In all experiments, slides were coded and were quantitatively or qualitatively analyzed by a blinded investigator. A minimum of three sections per animal was used in which all sections correspond to the same bregma coordinates in all animals. Quantification of PHi3+ cells in the adult SVZ and RMS was performed live under the epifluorescence microscope (Leica DMIRB). For quantifying Dcx+ cells in the adult RMS, images were acquired using Openlab software (Improvision) with a digital camera (Hamamatsu C4742-95) and the surface area occupied by a population of Dcx+ cells was measured using the lasso selection tool. Total Ki67+ and Ki67+/Dcx+ cells in the adult SGZ were quantified

after capturing images using an EVOS FL Auto 2 Imaging System (ThermoFisher Scientific). Quantification in all other experiments was performed after acquiring images using Zeiss LSM 710 laser scanning confocal microscope. Only cells that showed clear nuclear DAPI+ staining were included for analysis.

Real-time quantitative PCR (RT-qPCR)

For gene expression analysis, we used littermates that were derived from WT x WT or Sdy x Sdy breeding paradigm as described above. For *in vivo* experiments, postnatal littermates (N=3 per group) were repeatedly i.p. injected with saline or polyI:C. At P12, littermates were deeply anesthetized with an overdose of pentobarbitone and brains were dissected out and coronally sliced into 0.5mm slices using young mouse brain slicer matrix (Zivic instruments BSMYS005-1). Both striatal and septal SVZ were microdissected and SVZ tissues (N=3 per group) were pooled together and snap-frozen in dry ice and kept at -80°C until use. According to manufacturer instructions, total RNA samples were purified using RNeasy Mini Kit (Qiagen 74104) in which tissues were disrupted using Eppendorf micropestle (Sigma Z317314), lysates were homogenized using QIAshredder spin columns (Qiagen 79654) and DNA was removed with on-column DNase digestion set (Qiagen 79254). Equal amount of RNA samples per experiment were reverse transcribed into cDNA using the High-Capacity RNA-to-cDNA Kit (Applied Biosystems 4387406), and TaqMan gene expression master mix (Applied Biosystems 4369016) was used. For *in vitro* experiments, neurospheres that were incubated with pre-warmed saline or polyI:C (50µg/ml) for three hours were processed using Cells-to-CT 1-Step TaqMan Kit (Ambion A25603) in which 1.5µl cell lysates was used for all genes. We used littermates in all gene expression analysis in order to ensure that any detected response is likely due to the polyI:C treatment itself, and not due to any inherited variation from litter-to-litter. Since each experiment was replicated at least three times, thus animals were derived from at least three different litters.

The following inventoried TaqMan gene expression assays (Applied Biosystems) were used: Target genes *Dtnbp1* (Mm00458743_m1), *Tlr3* (Mm01207404_m1), *RelA* (Mm00501346_m1), *Sp1* (Mm00489039_m1), *β-actin* (Mm00607939_s1) and ribosomal 18S (*Rn18S*) (Mm03928990_g1). All experiments were run in 20µl reaction volumes in 96-well plates using StepOnePlus Real-Time PCR System (Applied Biosystems 4376600). Standard cycling conditions were run according to manufacture's protocol for each reagent. All measurements were performed in duplicates in which mean values of mRNA levels of the target genes were normalized to the geometric mean of β-actin and Rn18S[64] and relative gene expression of treated to non-treated controls per experiment was calculated using the $2^{-\Delta\Delta Ct}$ method[65]. No-template control was also run for each gene tested in all experiments to ensure no contamination of the RT-qPCR reactions.

Behavioural tests

Spontaneous locomotor activity

The spontaneous locomotion tests were carried out in 20 Acrylic chambers (AccuScan Instruments, Columbus, OH, USA) with the following dimensions (L×W×H= 17.5×10×26 cm). The activity chambers were equipped with infrared sensors in order to assess locomotion and the data were collected using the Versamax Software (version 4.0, AccuScan Instruments). On the test day between 9:00am to 11:00am, mice were placed in the activity chambers in a dimly lit room and their activities were monitored for two hours. The total horizontal distance (cm) traveled per mouse in 120 min was used for analysis.

Prepulse inhibition (PPI)

The PPI test was measured using the startle response system (SR-LAB, San Diego Instruments) consisting of multiple sound-attenuating cabinets, each equipped with a cylindrical animal enclosure and a small electric fan to provide ventilation and a background noise of 70 decibels (dB). Mice were familiarized to the enclosure for 5 min

before starting the tests. They were presented with noise pulses presented via a speaker placed directly above the mouse. Motion in response to the noise was detected via an accelerometer attached to the frame of the animal enclosure. The SR-LAB software was used to record the responses and also to control the noise pulse parameters.

Mice were first habituated to a startle (main) pulse of 120 dB in two trials (not included in analysis) followed by subsequent 40 trials in which the startle response to the main pulse was measured and analyzed. The main pulse was either presented alone or 100 milliseconds (ms) following prepulses of 30 ms duration with intensities of 3, 6, 9, 12 and 15 dB above the background noise. These prepulse intensities varied randomly between trials and each prepulse was presented five times. The average interval between trials was 15 seconds (s) (range 5-30 s). The startle responses were automatically determined by SR-LAB software. The percent PPI was calculated in the following formula: %PPI = [100 - (startle response to prepulse and pulse trials) ÷ (startle response to pulse alone trials) * 100].

Object recognition

An open-field chamber made of dark Plexiglas was used for the test. In a quiet room, mice were individually placed in this chamber (L×W×H: 45×45×45 cm) for 20 min each day for 3 days with two identical objects (toys, Dollar Store) in order to acclimatize them with the test environment. On the test day, the mice were placed in the chamber and allowed to explore two new identical objects for 5 min (familiarization phase), and were then placed back into their home cage for 5 min (retention time). During this time, the objects in the chamber were replaced with two new objects in which one of them had an identical shape to the objects used during the familiarization phase while the other had a novel shape. Finally, the mice were placed in the chamber and were allowed to explore the objects for 3 min

(testing phase).

The behavioural activities of mice were videotaped during both the familiarization and testing phases and were analyzed by investigators blinded to the genotype. The time was only calculated if a mouse was involved in object exploration based on the following criteria: 1) the mouse head was leaning towards the object and only within 2-3 cm away from it; 2) the mouse had at least one forepaw on the object and/or was sniffing or licking the object. Rodents naturally tend to explore novel rather than familiar objects in their environment. Therefore, object recognition memory was evaluated based on the increased time a mouse spent to explore a novel (TN) than a familiar (TF) object. This was determined using the following formula [Exploration ratio = TN ÷ (TF + TN)], in which a value significantly different from 0.5 (chance level) reveals the level of the recognition memory. The exploration time represents the total time of exploring novel or familiar objects during the testing phase.

Anxiety-like behaviour

Mice were tested in the elevated plus-maze (EPM) according to our previously described procedures [25]. The apparatus (made up of black-painted wood) consists of a plus-shaped maze with two closed and two open arms, each 50 cm × 5 cm in size raised 70 cm above the ground. The height of the closed arm walls is 15 cm. Animals were placed in the central arena, facing an open arm and allowed to explore the maze for 5 min. The session was recorded with a video camera positioned above the apparatus, and the recordings were scored for the time spent in the open and the closed arms. The ratio of time spent in the open arms vs. in the closed arms was analyzed as a measure of anxiety-like behaviour. The videos were analyzed for the number of entries into the open and closed arms as well as time spent in the open/closed arms.

Fear conditioning

We followed our previously described procedure with some modifications for the fear conditioning test [24]. Briefly, operant chambers (Kinder Scientific Instruments, Poway, CA) were equipped with a metal grid floor through which a shock of 0.5mA (unconditioned stimulus; US) is delivered and a son-alert to deliver an 85 dB tone (conditioned stimulus; CS). For CS/US conditioning trials, each animal was placed in the chamber for a total of 7 min. Two minutes after the start of the session, each mouse received two CS–US pairings with a fixed inter-trial interval of 2 min. The CS was presented for 30 s, and the US (0.5 mA) was presented during the last 2 s of CS presentation. The time the mouse spent freezing was calculated for each 30 s bin based on the automatic rest-time recording using "Motor Monitor" version 5.04 software supplied by the manufacturer.

To test for contextual fear conditioning (context as background), the mice were placed in the same shock chamber 24 h later without CS or US, and the percent of time spent freezing was calculated for 5 min. The auditory (cued memory) fear conditioning test was followed 1 h after the test for contextual conditioning in a novel operant box of the same size as the shock chamber, but with a non-shock floor and walls of different texture and color. After 3 min habituation period (pre-tone), mice were presented with the CS continuously for 3 min (post-tone). The percent of time spent freezing in pre-tone session as well as to the CS in the post-tone session was calculated for the whole three minutes.

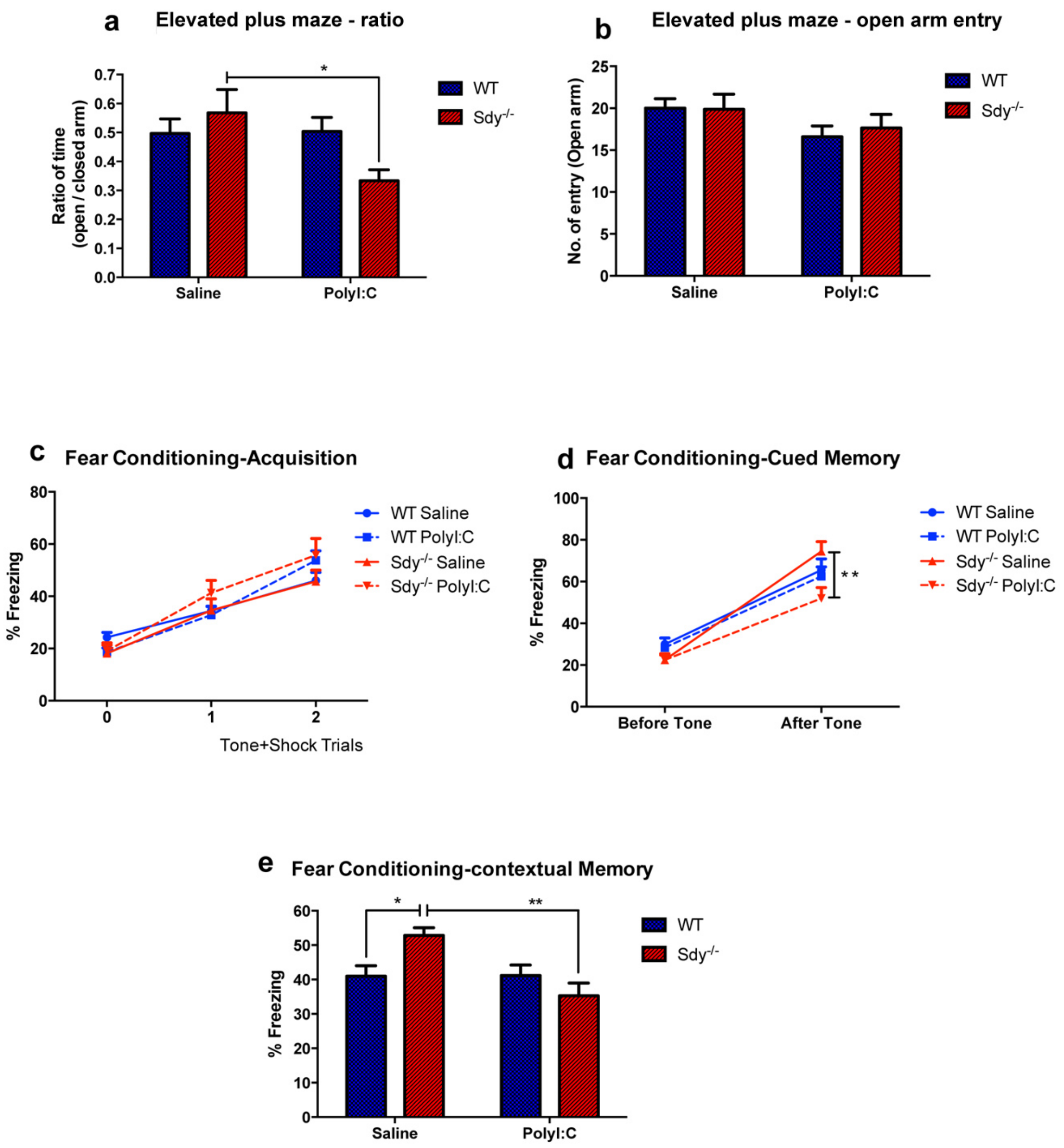

Supplementary Figure 1. Increased anxiety-like behaviour and impaired fear conditioning memory in adult Sdy mice given postnatal polyI:C. (a) The 0.5 ratio of time (open/closed arm) in WT saline mice indicates that the mice spent twice as much time in the closed arm versus the open arm. In comparison to the Sdy saline group, polyI:C injections to Sdy mice led to significantly increased anxiety-like behaviours as revealed by lower ratio of time spent in open arms vs. closed arms. (b) Number of entries in the open arms was not different in all groups. (c) All mice showed enhanced freezing response on day 1 of the fear acquisition trials. (d) In the cued memory test, Sdy polyI:C mice exhibited disrupted freezing response to conditioned stimulus (tone) in comparison to Sdy saline group. (e) In contextual memory test, Sdy mice showed abnormal freezing responses to shock context exposure 24 h following fear acquisition. All values are mean±s.e.m. of N=7-10 per group for (a,b) and N=8-11 per group for (c,d,e). Data were analyzed using two-way ANOVA for (a,b,e) or three-way ANOVA repeated measures for (c,d) with Tukey's post-hoc test. *P<0.05 and **P<0.01.

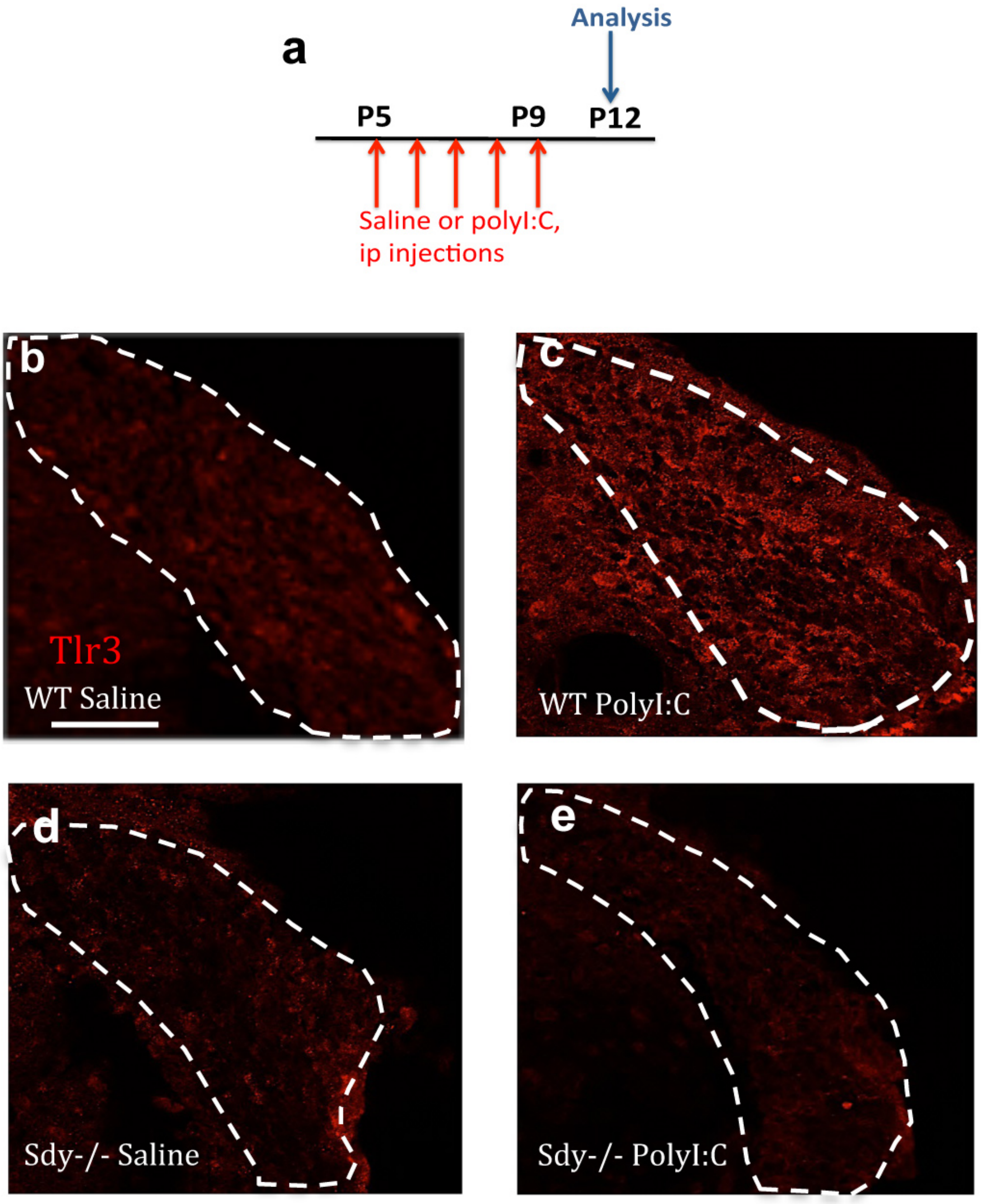

Supplementary Figure 2. Postnatal polyI:C induced Tlr3 protein expression in WT but not Sdy mice. (a) Design of i.p. injections in WT and Sdy mice, brains were collected for analysis at P12. The dorsolateral SVZ was analyzed for Tlr3 immunoreactivity. (b-e) Representative images of Tlr3 immunofluorescence in the SVZ (outlines) of WT and Sdy mice following saline or polyI:C injections. These were representative of N=2-3 per group. Scale bar 50 µm.

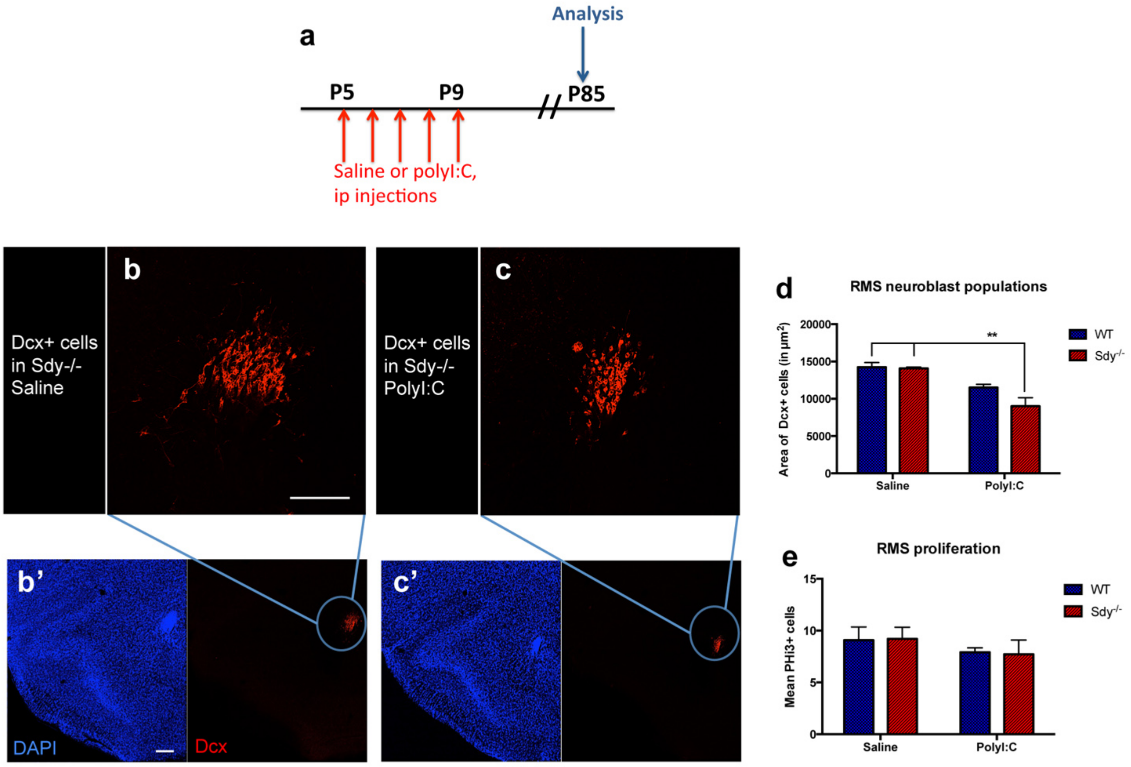

Supplementary Figure 3. Decreased neuroblast population, but not proliferation, in the RMS of adult Sdy mice given postnatal polyI:C injections. (a) Experimental design of postnatal saline or polyI:C injections to WT and Sdy mice, brains were collected for analysis in adulthood. (b-c') Representative images with the same anatomical coordinates illustrating the decrease in the RMS migratory neuroblasts in Sdy polyI:C group in comparison to Sdy saline group. b' and c' show the location of the RMS in low magnification images of DAPI and Dcx staining. (d) Reduced surface area of migratory Dcx+ neuroblasts in the RMS of adult Sdy mice given postnatal polyI:C in comparison to adult WT and Sdy controls. (e) Mean number of mitotic PHi3+ cells in the RMS was not different between all groups. All values are mean±s.e.m. of N=3 per group. Data were analyzed using two-way ANOVA with Tukey's post-hoc test. **P<0.01. Scale bars 100 µm.

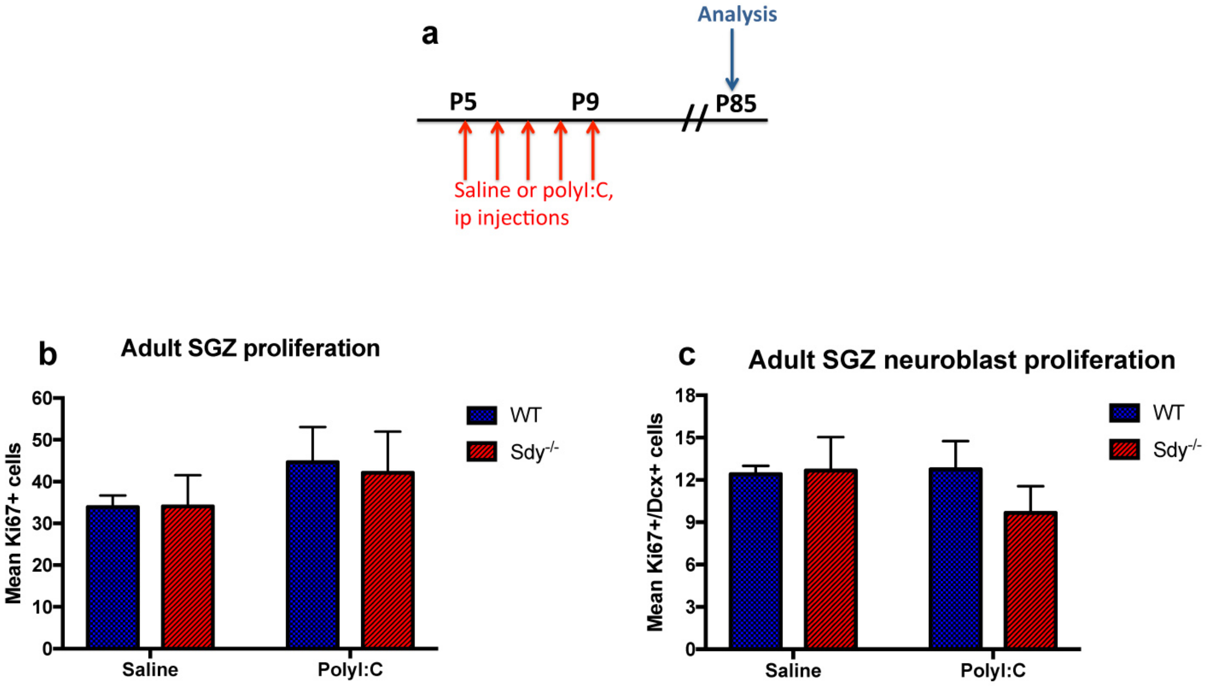

Supplementary Figure 4. Subgranular zone proliferation was not different to controls in adult Sdy mice given postnatal polyI:C injections. (a) WT and Sdy mice were injected with saline or polyI:C from P5-9 and brains were collected for analysis at P85 (adulthod). (b) The number of proliferative Ki67+ cells was not significantly different between the four groups. (c) The number of proliferative Ki67+/Dcx+ neuroblasts was also unchanged between all groups. All values are mean±s.e.m. of N=3 per group. Data were analyzed using two-way ANOVA.
